# The harpooning mechanism as evidenced in the oxidation reaction of the Al atom†
†Electronic supplementary information (ESI) available. See DOI: 10.1039/c7sc03314a


**DOI:** 10.1039/c7sc03314a

**Published:** 2017-11-02

**Authors:** Fangfang Li, Changwu Dong, Jun Chen, Jiaxing Liu, Fengyan Wang, Xin Xu

**Affiliations:** a Department of Chemistry , Collaborative Innovation Centre of Chemistry for Energy Materials (iChEM) , Shanghai Key Laboratory of Molecular Catalysts and Innovative Materials , Fudan University , Shanghai , 200433 , P. R. China . Email: fengyanwang@fudan.edu.cn ; Email: xxchem@fudan.edu.cn; b East China Sea Centre of Standard & Metrology (Technology) , SOA , Shanghai , 201306 , P. R. China; c College of Chemistry and Chemical Engineering , Collaborative Innovation Centre of Chemistry for Energy Materials (iChEM) , Xiamen University , Xiamen , P. R. China

## Abstract

The harpooning mechanism has long been proposed for elementary reaction dynamics involving metals. It is characterized by an initial electron transfer (ET) process from the metal to the oxidant molecule. For the titled reaction Al + O_2_, the ET distance can be predicted to be 2.6 Å by simply calculating the energy difference between the ionization energy of the Al atom and the electron affinity of the O_2_ molecule. Hereby we experimentally derived the maximum impact parameter *b*_max_ of 2.5 ± 0.2 Å for the titled reaction, in consistency with the predicted ET distance. This derivation of *b*_max_ was achieved by using the crossed molecular beam experiment at a collision energy of 507 cm^–1^ (*i.e.* 1.45 kcal mol^–1^) with a high resolution time-sliced ion velocity imaging detection of the state-selective AlO products based on the (1 + 1) resonance-enhanced multiphoton ionization. The small rotational constant of the AlO(X^2^Σ^+^) radical (*B*_e_ = 0.6413 cm^–1^) facilitated the formation of the AlO(*v* = 0) products in high rotational levels up to the energetically limited state, *N*_max_ = 52, with an almost zero velocity mapping. Hence, in this extreme angular momentum disposal case, the collisional orbital angular momentum *l* was nearly completely channeled into the product rotational angular momentum as a consequence of the conservations of energy and angular momentum, offering a reaction system that breaks the restriction of kinematically favored mass combination in order to obtain information on the impact parameters. The present study yields the first direct derivation of *b*_max_ from the maximum rotational level of products under the experimental condition with the recoil energy *E*′_T_ ≈ 0. This, in turn, provides solid evidence in supporting the harpooning mechanism.

## Abstract 2

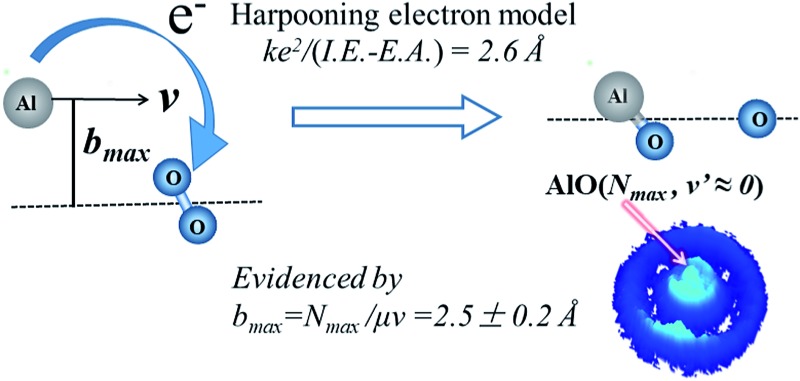
The harpooning model is firstly evidenced by the maximum impact parameter derived from AlO(*N*_max_) products with speed *v*′ ≈ 0 from the Al + O_2_ reaction.

## Introduction

The electron transfer (ET) process plays a fundamental role in many chemical systems.^1^ In the so-called harpooning mechanism, which has long been proposed as a primary process leading to metal atom reactions, the attacking metal atom tosses out its valence electron, hooks the oxidant molecule, and hauls it with the Coulomb force.^2^–^5^ By simply calculating the energy difference of the ionization energy (IE) of the metal atom and the electron affinity (EA) of the oxidant molecule, the ET distance can be predicted according to the formula:

1 *R*_C_ = *ke*^2^/(IE – EA)

where *k* is the Coulomb constant. This relates the size of the reaction cross section to the known energy.^5^ Despite the fact that the very large rate constants, corresponding to the very large reaction cross sections, measured for the alkali and alkaline earth metal atoms reacting with diatomic halogens could only be rationalized by invoking the mechanism of long-range electron transfer, and therefore harpooning, a quantitative comparison with eqn (1) has not been possible due to the large uncertainties in the measured reaction cross sections.^6^–^10^

For over five decades, no experimentally derived impact parameter *b*, defined as the distance of closest approach for a hypothetical undeflected trajectory of the colliding reagents,^1^ has been compared quantitatively with *R*_C_ to provide direct verification of eqn (1) for the harpooning distance. This is primarily a consequence of the difficulty encountered in a scattering experiment to determine the impact parameter *b*,^1^ although a few studies have been attempted along this line from the product rotational distributions on the kinematically favored mass combination, *e.g.* Ba + HI → BaI + H or Rb + HBr → RbBr + H.^11^–^15^ “In the absence of any detailed information on the recoil energy of the products”, as pointed out in previous research,^13^ it was, at best, an upper bound of the maximum impact parameter *b*_max_ that could be inferred by assuming the fulfillment of energy conservation. It is significant that in the case that the orbital angular momentum of the products lies in the opposite direction to their rotational angular momentum, considerations of merely rotational state distributions would have led to an overestimation of the total angular momentum of the collisional system, which, in turn, yielded an overestimation of the impact parameter.^16^ In these cases, it was necessary to acquire the information of the product orbital angular momentum, in conjunction with the rotational state-selective measurements. For the previously well-studied systems such as (Ba + HI) and (Rb + HBr),^3^–^7^ there is no experimental EA reported for HI or HBr. In fact, our calculations suggest that their corresponding anions are unstable with negative EA. Hence, one cannot explicitly calculate the electron transfer distance using eqn (1) to make a direct comparison.

Considering that the rotational excitation of the reactants is negligible in the crossed supersonic molecular beams, a very small exit relative translational energy *v*′_r_ will guarantee *l* ≈ *j*′, which corresponds to an extreme case of angular momentum disposal where nearly all of the reactant angular momentum *l* is channeled into the rotational angular momentum *j*′ of the product. Here *l* = *μbv*_r_, where *μ* is the reduced mass of the reactants and *v*_r_ is the collisional relative velocity. Then *b* would be derived from the product rotational level *j*′ at a negligibly small *v*_r_′. For the titled reaction Al + O_2_, the ET distance is calculated from eqn (1) as 2.6 Å using the experimental IE(Al) = 48 314 cm^–1^ (or 5.99 eV) and EA(O_2_) = 3549 cm^–1^ (or 0.44 eV),^17^ which can be used for an instructive comparison with the experimentally derived *b*_max_ to support the harpooning mechanism. Here, the adiabatic electron affinity is used, as the nuclei can adjust adiabatically with a small distance of about 0.15 Å (the difference between *r*_e_(O_2_) and *r*_e_(O_2_^–^)) during the transition from the non-ionic to the ionic state.^18^

The oxidation dynamics of the Al atom, an important process with potential applications in combustion and rocket propellants, have been studied in the crossed beam experiment by using a laser-induced-fluorescence (LIF) method and recently by the time-sliced ion velocity imaging technique.^19^–^26^ The reaction was found to be barrierless with Δ*H* = –1252 cm^–1^ (or –3.58 kcal mol^–1^).^23^ Due to the small rotational constant of the AlO product (*B*_e_ = 0.6413 cm^–1^), almost continuous rotational excitation has been observed.^19^–^26^ The speed distributions and angular distributions of the AlO products detected *via* resonance enhanced multiphoton ionization (REMPI) have been obtained at a collision energy of *E*_c_ = 1035 cm^–1^ (or 2.96 kcal mol^–1^) by Honma’s group.^26^ Compared to the study of Honma *et al.*,^26^ the improved energy resolution in our crossed beam and time-sliced ion velocity imaging setup^27^ has, as will be shown below, clearly resolved the two rotational states of the AlO products with a difference of rotational levels of 14 through the P and R branches, respectively, when recorded simultaneously at the same REMPI wavelength. This is necessary for determining the recoil energy distributions and the differential cross sections of the AlO products in a state well-resolved manner. In this work, we present direct evidence of the maximum impact parameter derived from the detailed information on the recoil energy of state-resolved AlO products formed from the titled reaction, Al(^2^P_1/2,3/2_) + O_2_(X^3^Σ–g) → AlO(X^2^Σ^+^) + O(^3^P_*J*_) at *E*_c_ = 507 ± 49 cm^–1^ (or 1.45 ± 0.14 kcal mol^–1^), which provides solid evidence in supporting the harpooning mechanism.

## Results

**Fig. 1:**
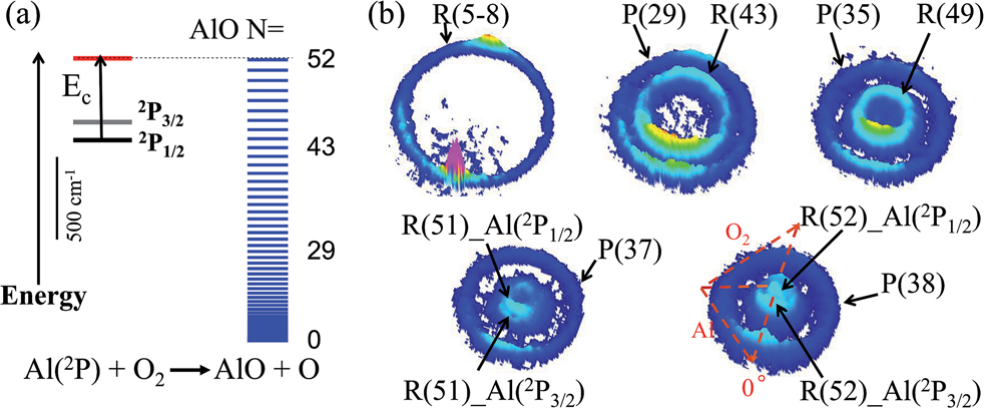
The energetics data for the reaction of Al(^2^P_*J*_) + O_2_(X^3^Σ–g) → AlO(X^2^Σ^+^) + O(^3^P_*J*_) at the collisional energy *E*_c_ = 507 ± 49 cm^–1^ (a); and the raw images of the AlO(X^2^Σ^+^, *v* = 0) products at various rotational levels *N* (b). The AlO products were rotational state-selective, ionized by (1 + 1) resonance-enhanced multi-photon ionization (REMPI) *via* the Δ*v* = 1 transition through the D^2^Σ^+^ intermediate state. The labels for rotational branches are shown in the slice images. The recorded inner (outer) ring with a slower (faster) speed corresponds to a higher (lower) *N*. With *N* approaching the maximum energetically available level, *N*_max_ = 52, the AlO(*N*) products from the reaction of spin–orbit coupling states of Al(^2^P_1/2_) and Al(^2^P_3/2_) were resolved. The Newton diagram is shown in the last image and the Al beam flying direction is defined as 0° in the centre-of-mass frame.

The energetics data of the Al(^2^P) + O_2_(X^3^Σ–g) reaction are shown in Fig. 1(a), and the available energy (*E*_c_ – Δ*H*) for the reaction system is approximately 1760 cm^–1^. An almost continuous rotational excitation of the AlO products to the energetically available limit is shown in blue in Fig. 1(a). The raw slice images of the AlO(X^2^Σ^+^) products at the vibrational ground state *v* = 0 are exemplified in Fig. 1(b), which were recorded at various wavelengths in the rotational state-selective (1 + 1) REMPI process. The label *N* in Fig. 1 represents the quantum number of the rotational angular momentum for AlO(X^2^Σ^+^) without considering the electronic spin coupling. Importantly, as compared to a previous study,^26^ this is a well-resolved state result when the two rotational levels of AlO(*N* and *N* + 14) are simultaneously probed at the same wavelength *via* P(*N*) and R(*N* + 14) transitions, respectively. The Newton diagram transforms the reactants’ velocity from the lab-frame to the center-of-mass (c.m.) frame. The reaction products will scatter around with a radius centered on the c.m. frame origin. In accordance with the conservation of energy, as *N* increases up to the highest energetically available level, the velocity mapped ring of the AlO(*N*) products contracts to a center spot representing an almost zero speed.

**Fig. 2:**
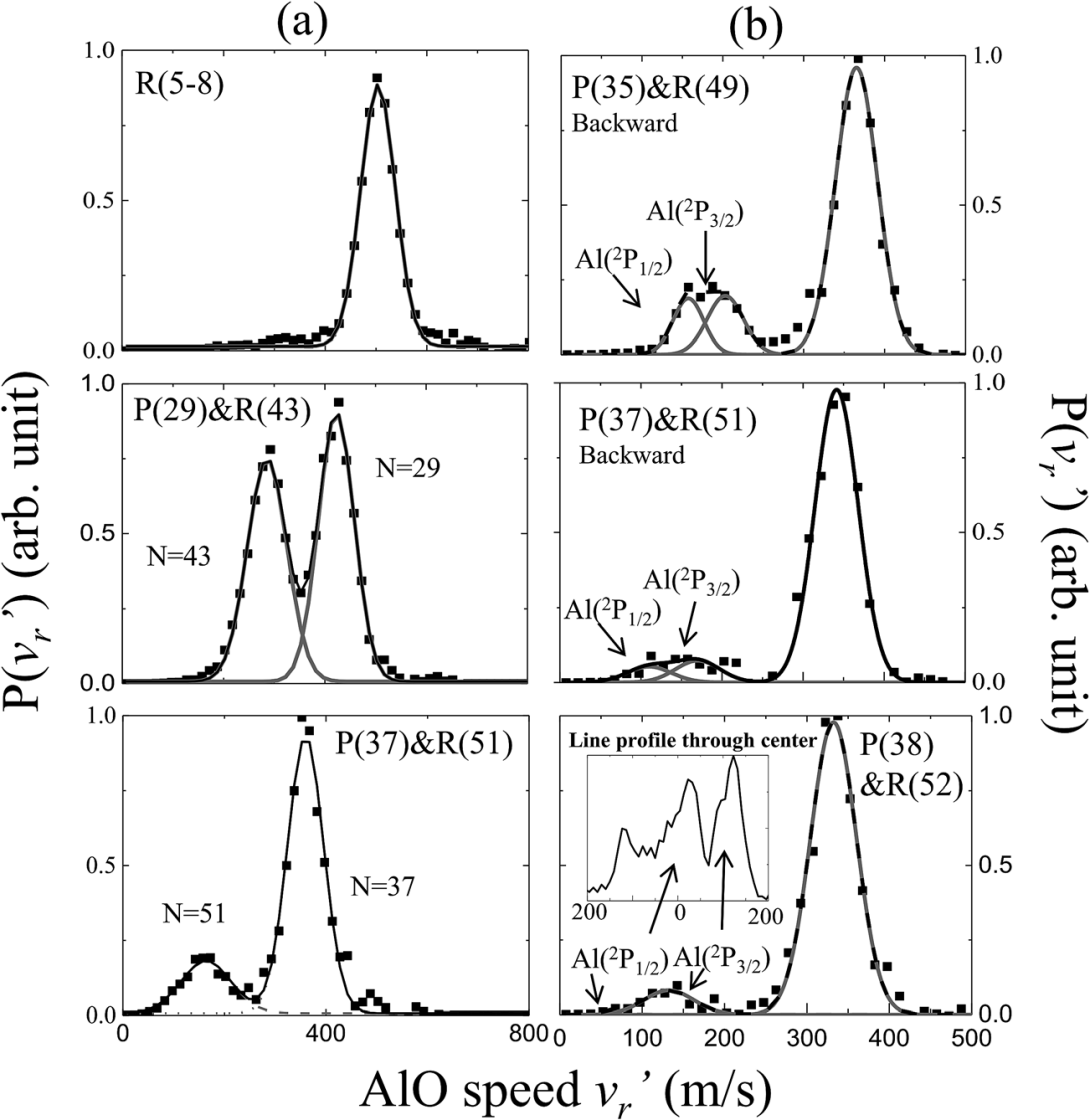
Normalized speed distributions of the AlO(X^2^Σ^+^, *v* = 0, *N*) products integrated over (a) the whole angular range and (b) the backward scattering direction from the slice images shown in Fig. 1. In accordance with the conservation of energy, the speed of the AlO(*N*) products in panel (a) decreases as *N* increases up to the maximum energetically available rotational level. In panel (b), the reactivity of different spin–orbit coupling states for Al(^2^P) could be resolved in the AlO products at higher rotational levels (*N* ≥ 49). In the last panel, the generated AlO(*N* = 52) from Al(^2^P_1/2_) and Al(^2^P_3/2_) can be clearly discerned from a simple line profile through the center of the raw image in the c.m. frame.

The speed distributions and angular distributions of the AlO products in different rotational states were extracted from the recorded slice images with the density-to-flux correction.^28^Fig. 2(a) shows the speed distributions P(*v*′_r_) of the AlO(X^2^Σ^+^, *v* = 0, *N*) products integrated over the whole angular range (0–360°). The speed distribution of AlO(*N* = 5–8) at low rotational levels shows the peak at ∼500 m s^–1^, converted to a total kinetic energy release (TKER) of about 1657 cm^–1^ in the AlO and O co-products. Neglecting a small rotational energy of about 19–46 cm^–1^ in the AlO products, almost all the available energy (1760 cm^–1^) of the system is channeled into the translational energy of the products.

As seen in Fig. 1(b) and 2(a), the P(29) and R(43) branches were recorded simultaneously at 244.543 nm for AlO(*v* = 0). The outer ring in the slice image with a faster speed of ∼425 m s^–1^ corresponds to a lower rotational level of *N* = 29 and the inner ring with a slower speed of ∼290 m s^–1^ corresponds to a higher rotational level of *N* = 43. The converted TKERs are 1197 and 557 cm^–1^ for *N* = 29 and 43, respectively, with an energy difference of 640 cm^–1^, which is consistent with the rotational energy space of 649 cm^–1^ between the two states. According to the conservation of energy, the energy separation between the peaks in the speed distributions should always be consistent with the corresponding internal energy difference of the AlO products between the recorded rotational levels.

As shown in Fig. 1(b) by the velocity mappings and Fig. 2(b) by the speed distributions, our experiments were able to distinguish the AlO products at *N* = 49, 51 and 52 generated from the oxidations of either Al(^2^P_3/2_) or Al(^2^P_1/2_), with a small energy difference of Δ*E* = 112 cm^–1^ due to spin–orbit coupling. The successful resolving of the spin–orbit reactivity of the Al(^2^P) atom in the AlO products again demonstrated the high resolution level achieved with our setup.^27^ As compared to the products at lower *N* with a larger translational energy, the AlO products at higher *N* have a greater possibility to resolve the reactivities of Al atoms in different spin–orbit coupling states. This can be easily understood, as a higher energy difference resolution Δ*E*/*E*_T_ is related to the products with a smaller translational energy *E*_T_. As clearly seen in Fig. 1(b), the signal of the AlO(*N* = 51 and 52) products in the c.m. frame corresponds to the ground state reaction of Al(^2^P_1/2_), while the signal with a larger radius ring corresponds to the excited state reaction of Al(^2^P_3/2_). Fig. 2(b) shows the speed distribution of the AlO products integrated over a narrow angular region (160–180°) for better resolution. From the integration of speed distributions in AlO(*N* = 51) and the population of Al states, we obtained a relative state reactivity of *σ*(Al(^2^P_1/2_))/*σ*(Al(^2^P_3/2_)) = 1.5 ± 0.3, which is half of the ratio obtained at a similar *E*_c_*via* LIF.^23^,^24^ A direct comparison between the present results and the previous LIF results shall be made with caution. We only observed the difference in the reactivity of two spin–orbit levels of Al (^2^P_1/2_ and ^2^P_3/2_) at high rotational levels of the AlO products, whereas LIF studies gave the reactivity difference in an all-states-integrated manner.

Notably, the AlO(*N* = 52) products with *v*′_r_ ≈ 0 from the oxidation of the ground state Al(^2^P_1/2_) become very weak in P(*v*′_r_). This is, however, reasonable as P(*v*′_r_) is obtained by the integration of the events in the raw images with the (*v*′_r_)^2^ factor taken into account over an angular range in the c.m. polar coordinate. When a line profile is taken through the center of the c.m. frame and roughly along the reactant relative velocity, the AlO(*N* = 52) products from Al(^2^P_1/2_) and Al(^2^P_3/2_) are clearly discerned as shown in the last panel in Fig. 2. This simple analysis sensitively sees the AlO product with *v*′_r_ ≈ 0, which lies at the center point of the c.m. system in the raw image. The above observation actually embodies another advantage of velocity mapping as shown in Fig. 1, where even the products with an almost zero speed distribution can be efficiently recorded.^28^

Consistent with the energetically available limit as shown in Fig. 1, the highest rotational level that can be observed is *N*_max_ = 52 in the vibrational ground state of the AlO products, which has a rotational energy of about 1751 cm^–1^. The higher rotational level of the AlO (*v* = 0, *N* = 53) product was also detected *via* the R(53) branch, as shown in Fig. S1.† A zero velocity of the AlO products from the Al(^2^P_3/2_) reactants was mapped and no reactive signal was observed for the ground state reaction.

**Fig. 3:**
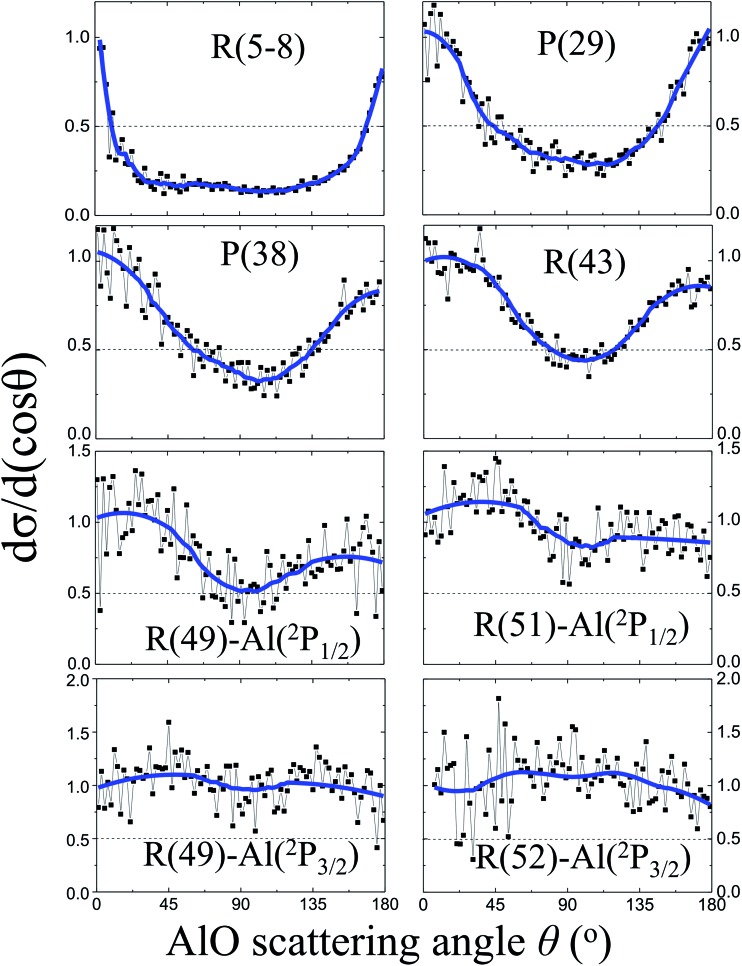
Normalized angular distributions d*σ*/d(cos *θ*) of the AlO(*v* = 0) products at various rotational levels from the oxidations of the Al(^2^P_1/2_ and ^2^P_3/2_) atoms. The raw data are shown in black and the smoothed fittings are shown in blue. The angular distributions of the AlO products display a sharp forward–backward peak at low *N* and a nearly isotropic distribution at high *N*. For AlO in high rotational levels, the reactivities of Al atoms in ^2^P_1/2_ and ^2^P_3/2_ states were resolved.

Fig. 3 shows the angular distributions d*σ*/d(cos *θ*) of the AlO products at various rotational states. At low rotational levels *N* = 5–8, the angular distribution of the AlO products is characterized with a sharp backward–forward peak at *θ* = 180° and 0°, respectively, and is close to the shape of 1/sin *θ*.^16^,^29^ As *N* increases and approaches the energetically limited value, more products were found to distribute in the sideways direction at *θ* = 90°. The reactivities of the Al atoms in the ^2^P_1/2_ and ^2^P_3/2_ states were resolved for the angular distributions of AlO in high rotational levels. Almost isotropic angular distributions were observed for the high rotational level *N* ≥ 49 from the excited state reaction involving Al(^2^P_3/2_) and for *N* ≥ 51 from the ground state reaction involving Al(^2^P_1/2_).

## Discussion

The results based on the present rotational state-selective speed distribution measurements, shown in Fig. 1 and 2, clearly show that the extreme case where the reactant orbital angular momentum was channeled into the product rotational angular momentum, *i.e. l* ≈ *j*′, was observed for the title reaction. As a consequence of the conservation of energy and the angular momentum, the almost zero speed of AlO(*N*_max_ = 52) suggests a negligible product orbital angular momentum.

The experimental proof for the establishment of the *l* ≈ *j*′ condition is of great significance for the direct experimental determination of the largest collision impact parameter *b*_max_. According to the equation 
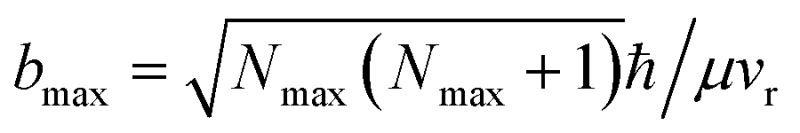
,^30^ where *ħ* is Planck’s constant divided by 2π, *b*_max_ is derived to be 2.5 ± 0.2 Å at the relative velocity *v*_r_ of 910 ± 50 m s^–1^ in the supersonic crossed beam experiment for the title reaction. It has to be emphasized that the experimentally derived *b*_max_ agrees well with the ET distance *R*_C_ ≈ 2.6 Å from the predicted value based on eqn (1). Thus, the present results provide solid experimental evidence in supporting the harpooning mechanism, *i.e.* the attacking Al atom uses its valence electron to “harpoon” the oxygen molecule, similar to the reaction mechanism proposed for an alkali atom to react with a halogen molecule.^5^

**Fig. 4:**
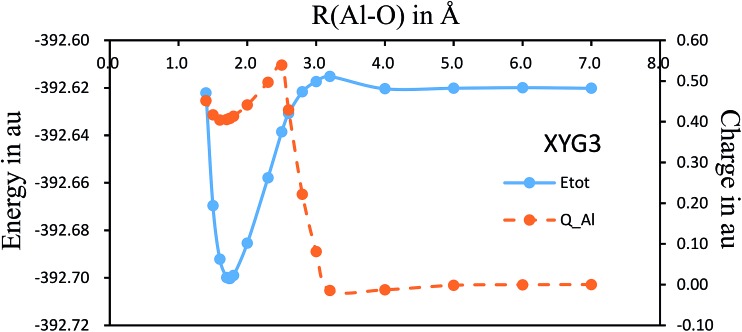
The potential energy (solid curve) section along the reactant channel studied in the *C*_∞v_ symmetry with a varying R(Al–O) distance from 7.0 to 1.4 Å while the R(O–O′) distance is being optimized. Along the energy profile, the results from the Mulliken charge analysis on Al (broken curve) are also shown.

There have been several preliminary theoretical studies for Al–O_2_.^31^–^33^ In particular, Ledentu *et al.* calculated a potential energy section along the reactant channel studied in the *C*_∞v_ symmetry with the distance between two oxygen atoms fixed at 1.2171 Å.^33^ We are constructing the global potential energy surface for the title reaction, where Fig. 4 shows the *C*_∞v_ potential energy curve with a varying R(Al–O) distance from 7.0 to 1.4 Å while the R(O–O′) distance is being optimized. Along this energy profile, we also show the results from charge analysis. Indeed, a neutral-ionic avoided crossing region exists, around which a sudden electron transfer from Al to O_2_ occurs. The point with the maximum charge transfer corresponds to R(Al–O) = 2.5 Å, while the co-linear AlOO′ complex has R(Al–O) = 1.735 Å with R(O–O′) = 1.317 Å. Hence, it can be envisioned from Fig. 4 that the electron is first transferred, hooking the ion pairs through the coulombic force, which helps the reaction to proceed further to form an AlOO′ complex before complete dissociation of O–O′. Currently, we are constructing the global potential energy surface for the title reaction to examine the detailed dynamics.

For the barrierless title reaction,^21^ the opacity function is a step function and the reaction probability is independent of the impact parameter when *b* ≤ *b*_max_. Thus the reaction cross section *σ* = π*b*_max_^2^ is estimated to be 19.6 Å^2^ at the collision energy of this study. The resultant rate constant *k*(*v*_r_) = *v*_r_*σ* is 1.8 ± 0.02 × (10^–10^ cm^3^) per molecule per s with a narrow relative velocity of 910 ± 50 m s^–1^, which can be compared to the thermal rate constant *k*(*T*) = (1.5–1.7) × 10^–10^ cm^3^ per molecule per s in the temperature range of *T* = 300–600 K with a mean relative velocity in the range of 660–930 m s^–1^.^21^ The nearly constant value of *k*(*T*) in the temperature range is partly caused by a negative dependence of the reaction cross section on the collision energy.^21^,^34^ In relation to *k*(*v*_r_), *k*(*T*) could be loosely written as *k*(*T*) = *v*_r_*σ*(*v*_r_)_*T*_ ≈ *v*_r_*σ*(*v*_r_)*_T_*, where the brackets denote the average over a thermal distribution of the relative velocity at temperature *T*. Thus, the comparable values of *k*(*v*_r_) and *k*(*T*) provide credible evidence for the impact parameter of 2.5 Å obtained here.

The observed negative dependence of the reaction cross section on the collision energy for the title reaction^21^,^34^ is understandable. If the reactant passed very quickly, the attractive force, *i.e.* the coulombic force in the harpooning mechanism, would not be enough to turn the reactant around to let the reaction happen. In other words, with the increase of the collision energy *E*_c_, the centrifugal energy E_c_*b*^2^/*R*^2^ will contribute increasingly as a repulsive energy at the corresponding impact parameter. Thus in order to get an accurate electron transfer distance to compare with the harpooning model, one needs to minimize the dynamics effect of the centrifugal energy and measure the maximum impact parameter at a low collision energy, which leaves enough time for long-range attractive interactions.^1^ This is indeed the setting of the present experiment.

The experimental observation for the dependence of angular distributions d*σ*/d(cos *θ*) on the product rotational angular momentum *N* can be well interpreted by the model of the angular momentum disposal in a complex-forming reaction as proposed by Kim and Herschbach.^16^,^29^ As clearly shown in Fig. 3, the angular distribution displays a trend changing from the backward–forward peaking to an isotropic distribution as the products have been rotationally excited to a high rotational level. At low *N*, the sharp backward–forward angular distributions suggest that the corresponding plane of collision is conserved with *l* ≈ *l*′.^16^,^29^ On the other hand, the nearly isotropic distribution at high *N* implies the effect of nonplanar scattering where a significant angular momentum appears as a product rotation with respect to the random axis with *l* ≈ *j*′.^16^,^29^

Experiment–theory comparisons in reaction dynamics always involve averaging over all possible impact parameters. This is true even for a quantity such as the state-resolved differential cross section. In this work, the measurement of the maximum impact parameter will assist and test the quantum chemical calculations to explore the oxidation dynamics of the Al atom in a particular range of impact parameters. Further experimental information regarding the harpooning mechanism can be obtained by studying the stereochemistry of collisions, wherein one can determine the relationship between the non-spherical orbital and the product scattering angle.^35^–^40^ A more detailed analysis of differential cross sections from various reactant alignment conditions would offer more clear evidence to establish the reaction mechanism and to allow a 3D visualization of how the chemical transformation takes place.

## Methods

The experimental apparatus (Fig. S2†) used in this work has been described elsewhere,^28^ and the experimental techniques involved the laser-ablation and the crossed-beam setup combined with time-sliced ion velocity imaging detection.^27^,^28^,^41^–^44^ Only a brief account is provided here. The supersonic aluminum atomic beam (speed ∼ 530 m s^–1^), produced by laser vaporization of an Al metal rod (97% Al, Alfa) with Ar as the carrier gas, intersected at 90° with the supersonic molecular beam of the pure oxygen molecule (speed ∼ 740 m s^–1^) in the centre of the ion optics. The Al(^2^P) atomic beam was characterized by (1 + 1) REMPI *via* the Al(^2^D) intermediate state, for Al(^2^P_1/2_) at 308.305 nm and Al(^2^P_3/2_) 309.360 nm, respectively. With Ar as the carrier gas, we obtained a ratio of Al(^2^P_1/2_)/Al(^2^P_3/2_) = 0.47 ± 0.03 (one standard deviation per error bar) (Fig. S3†). The AlO products were detected by (1 + 1) REMPI *via* the D^2^Σ^+^ – X^2^Σ^+^ transition in the wavelength region of 243–245 nm.^26^ The probe laser beam was generated from a Continuum Sunlite OPO/OPA laser pumped by a Nd:YAG laser at 355 nm with a 5–9 ns pulse width. A pulse energy of 1–3 mJ was used with a cylindrical lens (*f* = 500 mm) with the focus direction along the extraction axis. The probe laser had a large spot size of approximately 10 mm in the unfocused direction to cover the molecular-beam-crossed region in the collision plane. The spot size of the probe laser suggests that the time accumulation of the reactive collision events is about 10 μs and this accumulation period narrows the relative velocity to a specified *E*_c_.

The product ions with the same velocity were accelerated and projected upwards by the ion optics onto the same point on the position sensitive detector. The detector is composed of two micro-channel plates (75 mm 60 : 1 10 μm pores and 12 μm pitch, Photek) and one Phosphor Screen (P43, Photek). Slice images were finally recorded by applying a 30 ns gate pulse onto the detector. To remove the AlO background in the Al atomic beam ablated from the surface of the Al rod due to the oxide contamination, an active subtraction scheme was employed routinely for the images taken with the O_2_ beam on and off in turn for all data acquisition. In fact, the AlO background in the Al beam has the velocity mapped along the direction of the flying Al beam, and is just outside of the flying area of the reactive products with maximum kinetic energy. Thus the AlO background did not affect the distributions of products in the various rotational levels from which impact parameter information was obtained.

The calculations were performed using the XYG3 doubly hybrid density functional.^45^ With systematic and comprehensive tests, it has been concluded that XYG3 is very accurate for main group chemistry.^46^ Recently we have shown that XYG3 can describe the simplest tri-atomic chemical reaction, H + HH′ → H′ + H_2_, in a state-to-state level well,^47^ and can provide accurate potential energy surfaces for a series of hydrogen abstraction reactions^48^ for quantum dynamics applications. The basis set used is 6-311+G(3df,2p).

## Conflicts of interest

There are no conflicts to declare.

## Supplementary Material

Supplementary information
