# Supplementary material for: The harpooning mechanism as evidenced in the oxidation reaction of the Al atom
Source: Chem Sci. 2017 Nov 2;9(2):488–94. doi: 10.1039/c7sc03314a (PMC5868079; doi:10.1039/c7sc03314a)
Supplement: Supplementary file 1 [file SC-009-C7SC03314A-s001.pdf]

Electronic Supplementary Material (ESI) for Chemical Science.
This journal is © The Royal Society of Chemistry 2017

Electronic supplementary information for

The harpooning mechanism as evidenced in the oxidation reaction of the Al atom

Fangfang Li[1†], Changwu Dong[1,2†], Jun Chen[1,3], Jiaxing Liu[1], Fengyan Wang[1], and Xin Xu[1]**

***Corresponding Authors. Email: fengyanwang@fudan.edu.cn and xxchem@fudan.edu.cn**

This PDF file includes:

Figs. S1-S3

Reference

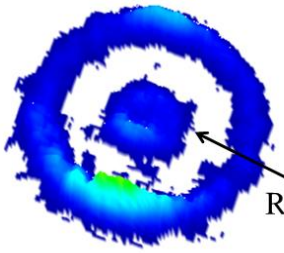

Fig. S1 The slice image of the AlO products detected via the (1+1) resonance enhanced multiphoton ionization at 244.947 nm. The inner ring corresponds to AlO(v = 0, N = 53) from the reaction of the excited Al($^2P_{3/2}$) spin-orbit state. No AlO product was observed from the reaction of the ground spin-orbit state of Al($^2P_{1/2}$), which reconfirmed that the maximum energetically available level for the ground state reaction is N_{max} = 52. Because the probe wavelength was overlapped with the wavelength for P(1) and R(15) branches for the AlO(v = 1) products, the AlO(v = 1, N = 1 and 15) products were also detected and the corresponding signals overlapped with the outer ring of the image for the AlO(v = 0, N=39) channel.

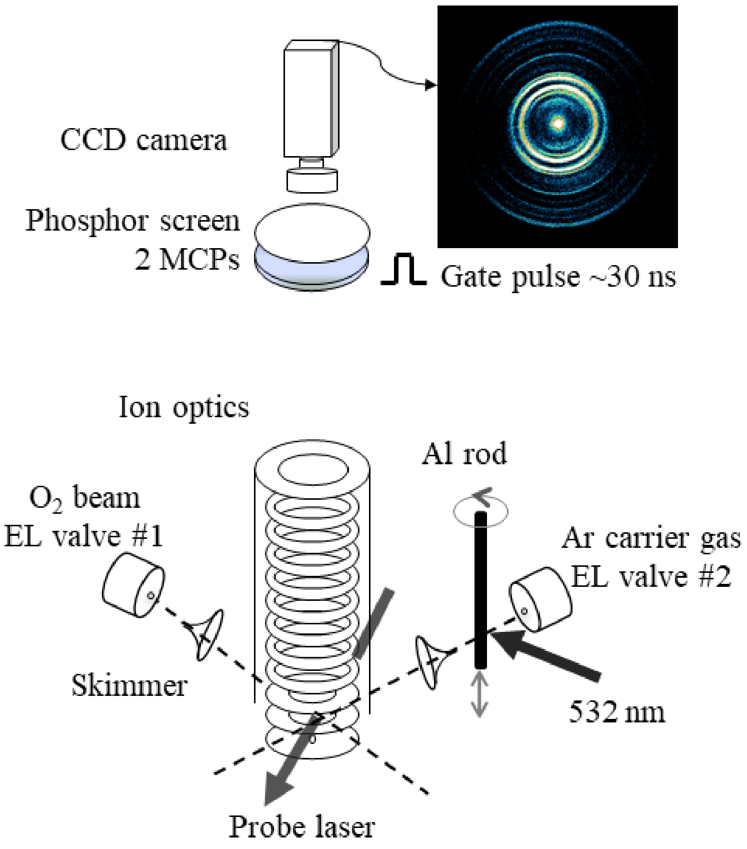

Fig. S2 A schematic view of our crossed-beam apparatus for studying the title reaction (adapted from Ref.S1). The geometries for crossed molecular beam and the probe laser are shown in the figure. The calibration for velocity mapping was done with the speed distribution of O^+ ions from the multiphoton dissociation dynamic of O_2 molecules at 225 nm. The image of O^+ ions is shown on the top.

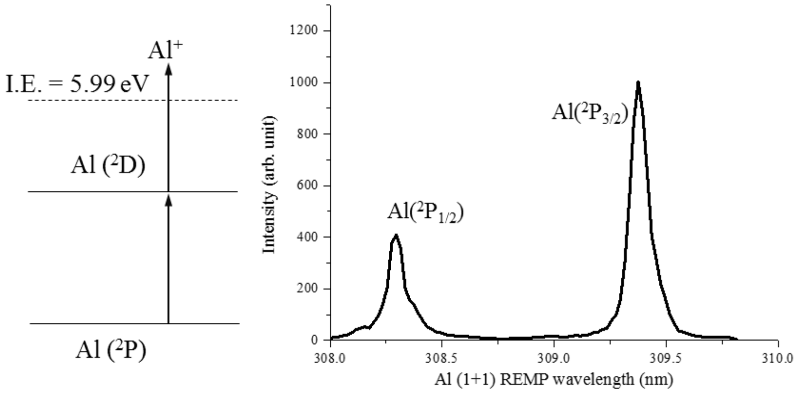

Fig. S3 Schematics of (1+1) REMPI for Al(^{2}P) detection and the recorded spectra of the Al(^{2}P) spin-orbit states from the Al reactant beam in the wavelength region of 308 – 310 nm. The spectra were the averaged results of three times repeated experiments. A ratio of Al($^2P_{1/2}$)/Al($^2P_{3/2}$) = 0.47 ± 0.03 was obtained (one standard derivation per error bar).

Reference

s1. C. W. Dong, J. X. Liu, F. F. Li and F. Y. Wang, *Chin. J. Chem. Phys.*, 2016, **29**, 99-104.
